# Chemoreception of Mouthparts: Sensilla Morphology and Discovery of Chemosensory Genes in Proboscis and Labial Palps of Adult *Helicoverpa armigera* (Lepidoptera: Noctuidae)

**DOI:** 10.3389/fphys.2018.00970

**Published:** 2018-08-07

**Authors:** Mengbo Guo, Qiuyan Chen, Yang Liu, Guirong Wang, Zhaojun Han

**Affiliations:** ^1^Education Ministry Key Laboratory of Integrated Management of Crop Diseases and Pests, College of Plant Protection, Nanjing Agricultural University, Nanjing, China; ^2^State Key Laboratory for Biology of Plant Diseases and Insect Pests, Institute of Plant Protection, Chinese Academy of Agricultural Sciences, Beijing, China

**Keywords:** *Helicoverpa armigera*, mouthparts, sensilla, transcriptome, chemosensory genes

## Abstract

Siphoning mouthparts, consisting of proboscis and labial palps, are the exclusive feeding organs and important chemosensory organs in most adult Lepidoptera. In this study, the general morphology of the mouthpart organs and precision architecture of the proboscis was described in adult *Helicoverpa armigera.* Three major sensilla types with nine subtypes including three novel subtypes were identified. The novel sensilla styloconica subtype 2 was the only one having a multiporous structure, which may play olfactory roles. For further understanding of the chemosensory functions of mouthpart organs, we conducted transcriptome analysis on labial palps and proboscises. A total of 84 chemosensory genes belonging to six different families including 4 odorant receptors (ORs), 6 ionotropic receptors (IRs), 7 gustatory receptors (GRs), 39 odorant binding proteins (OBPs), 26 chemosensory proteins (CSPs), and 2 sensory neuron membrane proteins (SNMPs) were identified. Furthermore, eight OBPs and six CSPs were identified as the novel genes. The expression level of candidate chemosensory genes in the proboscis and labial palps was evaluated by the differentially expressed gene (DEG) analysis, and the expression of candidate chemosensory receptor genes in different tissues was further investigated by quantitative real-time PCR (qRT-PCR). All the candidate receptors were detected by DEG analysis and qRT-PCR, but only a small part of the OR or IR genes was specifically or partially expressed in proboscis or labial palps, such as *HarmOR58* and *HarmIR75p.1*, however, most of the GRs were abundantly expressed in proboscis or labial palps. The reported CO_2_ receptors such as *HarmGR1*, *GR2*, and *GR3* were mainly expressed in labial palps. *HarmGR5*, *GR6*, and *GR8*, belonging to the “sugar receptor” clade, were mainly expressed in proboscis or antenna and were therefore suggested to perceive saccharide. The results suggest that the mouthparts are mutually cooperative but functionally concentrated system. These works contribute to the understanding of chemical signal recognition in mouthpart organs and provide the foundation for further functional studies.

## Introduction

As the foremost center for sensing and food ingestion, the head of most insects possesses several sophisticated organs, including antenna and mouthpart appendages. These organs play crucial roles in almost all activities conducted by insects, including detecting host plants, feeding, recognizing mates, or locating oviposition sites. Antennae are considered to be the most important multimodal sensory organs, and they contain a huge number of sensilla for perceiving not only odorants but also flavors, carbon dioxide, and mechanical stimulation (Keil, 1999). The mouthparts act as the exclusive organ for feeding, and they also have functions in chemoreception.

Morphology and evolutionary biology of the mouthparts have been well studied previously (Kristensen, 1984; Krenn et al., 2005; Nielsen and Kristensen, 2007; Lehnert et al., 2016). The majority of adults in Lepidoptera suborder Glossata possess typical siphoning mouthparts: a proboscis adapted to their feeding properties and a pair of labial palps, together with vestigial maxillary palps. As a feeding device, the proboscis consists of the pair of maxillae galeae, which are equipped with various sensilla. The capillary construction is generated by joining the two galeae together, which can then be used for sucking liquids. Various types of sensilla have been found on the proboscis, and there are significant differences among species (Krenn et al., 2001; Xue and Hua, 2014; Lehnert et al., 2016; Xue et al., 2016). The labial palps are located on each side of the proboscis and typically possess two or three segments. The role of labial palps in CO_2_ sensing has been demonstrated in several moth species such as *Pieris rapae, Manduca sexta, Bombyx mori, Mythimna separata*, and *Helicoverpa armigera* (Lee et al., 1985; Kent et al., 1986; Stange, 1992; Zhao et al., 2013; Dong et al., 2014).

Reception of chemical signals is mediated by three families of chemoreceptors (OR, IR, and GR) with the assistance of OBP, CSP, or SNMP in the sensilla (Benton et al., 2007; Jin et al., 2008; Leal, 2013; Fleischer et al., 2017; Pelosi et al., 2017). The peripheral perception of chemosensory stimulants was mediated by several families of olfactory proteins including odorant-binding proteins (OBPs), chemosensory proteins (CSPs), odorant receptors (ORs), gustatory receptors (GRs), ionotropic receptors (IRs), and sensory neuron membrane proteins (SNMPs). The stimulants diffuse into the cavity of sensilla through micropores on the cuticular surface and then are captured by two major families of small soluble proteins such as OBPs and CSPs (Vogt et al., 1991; Pelosi and Maida, 1995; Angeli et al., 1999; Pelosi et al., 2006, 2017). Then they are moved to the dendrite membrane of chemo-sensing neurons, where several families of the transmembrane receptors (ORs, GRs, and IRs) are expressed (Benton et al., 2009; Wang et al., 2010; Ai et al., 2013; Liu et al., 2013; Cao et al., 2016; Ning et al., 2016; Xu et al., 2016). The neurons are activated by stimulants, and then the olfactory signal is transmitted by action potentials to the primary olfactory processing center, that is, the antennal lobes (ALs) (Hansson and Christensen, 1999). The signals are further processed across multiple levels of downstream neural pathways, finally provoking a corresponding behavioral response (Hansson, 1995; Leal, 2013; Riffell and Hildebrand, 2016; Fleischer et al., 2017).

*Helicoverpa armigera* is one of the most damaging and highly polyphagous pests in China and many other regions all over the world; the larvae populate more than 120 plant species such as cotton, tomatoes, and maize and have caused serious economic losses (Firempong and Zalucki, 1989; Wu and Guo, 1997). To date, much progress has been made in morphological studies and in identifying chemosensory genes in antennae of *H. armigera* (Liu et al., 2012; Liu N.Y. et al., 2014; Zhang et al., 2015; Chang et al., 2016). For the mouthpart organs, the fine structure of labial palps has been studied carefully by Zhao et al. (2013). Each of the labial palps consists of three segments that are covered with scales. The third segment of labial palp possesses an invaginated bottle-shaped structure called the labial-palp pit organ (LPO). Almost 1,200 sensilla have been found in each LPO. Hair-shaped and club-shaped sensilla were found on the upper and lower half of the pit, respectively. Although the general structure of the proboscis in *H. armigera* has been reported previously, only a few sensilla types were described, perhaps due to the small number of sensilla or the resolution ratio of images.

Our previous studies have identified 66 ORs, 21 IRs, 33 OBPs, 24 CSPs, and 2 SNMPs mainly in antenna through transcriptome sequencing, and Xu et al. (2016) reported 197 GRs based on the genome and transcriptome sequencing (Liu et al., 2012; Liu N.Y. et al., 2014; Li et al., 2015; Zhang et al., 2015; Xu et al., 2016; Chang et al., 2017). Abundant chemosensory genes have been identified in the antennal transcriptome of numerous insects (Gong et al., 2007; Grosse-Wilde et al., 2011; Bengtsson et al., 2012; Zhang et al., 2015, 2016; Wang et al., 2017), but systematic gene excavation in mouthpart organs has not been done. Therefore, we were interested in determining how many types of chemosensory sensilla are in mouthpart organs and whether the mouthpart organs express abundant chemosensory proteins as in the antennae.

For a better understanding on the morphology of mouthparts, the microstructure was determined using an electron microscope scan experiment in this study. Further, we systematically investigated the chemosensory protein families in the labial palps and proboscis by transcriptome sequencing. The differentially expressed gene (DEG) analysis of all the candidate chemosensory genes and qRT-PCR analysis of candidate chemosensory receptor genes were performed to investigate the gene expression levels. This work contributes to the morphological and molecular studies on the mouthpart organs of *H. armigera*.

## Materials and Methods

### Insect Rearing and Tissue Collection

The larvae of *H. armigera* were fed with an artificial diet and kept in the conditions of 16:8 h (light:dark) photoperiod, 27°C ± 1°C and 50–60% RH at the Institute of Plant Protection, Chinese Academy of Agricultural Sciences, Beijing, China. Male pupae were kept separately from females. The moths were fed on 10% honey water after emergence. For transcriptome sequencing, the proboscis and labial palp were collected separately from the 1- to 3-day-old moths and then stored in liquid nitrogen immediately until they were used for experiments.

### Scanning Electron Microscopy and Sensillum Characterization

The proboscises from 1-day-old moth of eight females and eight males were tweezered from the base carefully and then were dehydrated in a series of ethanol (70, 80, 95, and 100%). After drying in a critical point drier (LEICA EM CPD), antennae were sprayed with gold (EIKO IB-3). The samples were then glued onto SEM stubs using a double graphite adhesive tape. Scanning was performed on a Hitachi SU8010 scanning electron microscope (Hitachi, Tokyo, Japan) at 10 kV. Sensillum types were characterized based on the description in the review about the proboscis sensillum types of the Lepidoptera by Faucheux (2013). The images were adjusted using Adobe Photoshop CS6 (Adobe Systems), but only the brightness and contrast. All figures were assembled in Adobe Illustrator CS5 (Adobe Systems).

### RNA Extraction and Transcriptome Sequencing

The total RNA of proboscis and labial palps was extracted following the manufacturer’s instructions using TriZol reagent (Invitrogen, Carlsbad, CA, United States). The quality of RNA was measured using an Agilent 2100 Bioanalyzer (Agilent Technologies, Santa Clara, CA, United States) and a NanoDrop ND-2000 spectrophotometer (NanoDrop products, Wilmington, DE, United States). One microgram of total RNA of each tissue (male and female mixtures) was used for generating a cDNA library, respectively. Construction of the cDNA library and Illumina HiSeq 2000 (Illumina, San Diego, CA, United States) sequencing was performed at the Beijing Genomics Institute (BGI, Shenzhen, China). The insert sequence length was around 200 bp, and these libraries were paired-end sequenced using PE100 strategy.

### Assembly and Functional Annotation

After filtering low-quality reads, trimming low-quality nucleotides at each end, and removing 3′ adaptors and poly-A/T tails from the raw reads, *de novo* assembly was conducted using Trinity. The clean reads of the proboscis and labial palps were fed to Trinity. The Trinity outputs were clustered by TGICL (Pertea et al., 2003). Unigene annotation was performed by NCBI BlastX against the database of non-redundant (nr) and SwissProt protein database with the *E*-value < 1*e*-5.

### Identification of Chemosensory Genes

Putative chemosensory genes of six families (ORs, IRs, GRs, OBPs, CSPs, and SNMPs) were screened with a series of strategies. Sequences were extracted using chemosensory gene keywords by running Perl scripts against assembly and annotation files of transcriptomes on the server. After removing redundant sequences, the genes were further confirmed by BlastX against a local non-redundant database under the *E*-value < 1*e*-5. The ORFs of all genes were predicted using the ExPASy server^1^ based on the BlastX best hit result (Gasteiger et al., 2003). Putative N-terminal signal peptides of OBPs and CSPs were predicted using the SignalP 4.0 server^2^ with default parameters (Petersen et al., 2011).

### Sequence and Phylogenetic Analysis

Alignments of amino acid sequences were performed in MAFFT^3^. The phylogenetic trees of chemosensory genes were constructed using RAxML version 8 with the Jones–Taylor–Thornton amino acid substitution model (JTT) (Stamatakis, 2014), and 1000 bootstrap replicates were run to assess the node support. The OBP phylogenetic tree was constructed using a total of 134 OBPs of four Lepidoptera species: 45 from *H. armigera* including 39 identified in our dataset, 26 from *Spodoptera littoralis*, 30 from *H. assulta*, and 33 from *Bombyx mori* (Gong et al., 2009; Jacquin-Joly et al., 2012; Liu et al., 2012; Zhang et al., 2015; Chang et al., 2017). For CSPs, 82 sequences were used including 30 from *H. armigera* (including 26 from our data), 15 from *H. assulta*, 21 from *S. littoralis*, and 16 from *B. mori* (Gong et al., 2007; Jacquin-Joly et al., 2012; Liu et al., 2012; Li et al., 2015; Zhang et al., 2015; Chang et al., 2017). The phylogenetic tree of SNMPs was constructed using 21 sequences of 11 species from Diptera and Lepidoptera. Sequences of novel *HarmOBPs* and *HarmCSPs* are shown as **Supplementary File S1**.

### DEG Analysis

Differentially expressed gene analysis between proboscis and labial palps was conducted using a mapping-based expression profiling analysis according to the strategies described by Wang et al. (2017). The expression levels of chemosensory genes (ORs, IRs, GRs, OBPs, CSPs, and SNMPs) were estimated by fragments per kilobase million (FPKM) values (Trapnell et al., 2010). The heat map of differential gene expression between male antennae and female antennae in both species was generated by iTOL software^4^.

### qRT-PCR Analysis and Statistical Analysis

The total RNA of four tissues including the antenna, proboscis, labial palps, and legs was extracted using TRIzol reagent (Invitrogen, CA, United States) according to the manufacturer’s protocol. The cDNA of each tissue reverse transcribed from 1 μg total RNA using revert aid first-strand cDNA synthesis kit (Thermo Scientific, Waltham, MA, United States). The mRNA expression level of each gene (ORs, IRs, and GRs) was examined by qRT-PCR using GoTaq^®^ qPCR Master Mix (Promega, WI, United States) and normalized by a reference gene *HarmActin*. PCR amplification was conducted using a ABI 7500 Real-Time PCR System (ABI, Vernon, CA, United States). The total volume of each reaction was 20 μL, which contains 10 μL of GoTaq qPCR Master Mix, 1 μL of each gene specific primer (10 μM), 2 μL of cDNA, and 6 μL of RNase-free water. The PCR cycling condition was set based on the manufacturer’s recommendations as follows: 95°C for 2 min, 40 cycles of 95°C for 15 s, and 60°C for 50 s. A melting curve analysis was performed to confirm the amplification efficiency of each pair of primers. The primers were listed in **Supplementary Table S2**. The expression level of each was quantified using the comparative *C_rmT_* method (Schmittgen and Livak, 2008). The Δ*C*_T_ was obtained by subtracting the *C*_T_ of *HarmActin* in a same tissue from that of a specific gene. The relative expression of each gene was evaluated by the values of 2^-ΔΔCT^, and the ΔΔ*C*_T_ was normalized by the mean Δ*C*_T_ of at least three repetitions in one tissue, which has the smallest Δ*C*_T_. The column diagram of each gene was constructed by GraphPad Prism 6 (GraphPad software Inc., La Jolla, CA, United States). The differences of expression among tissues and sexes were analyzed by one-way ANOVA and followed by Duncan’s test (*P* < 0.05) using SPSS 22 (SPSS Inc., Chicago, IL, United States).

## Results

### Morphological Structure of the Mouthpart Organs

**FIGURE 1:**
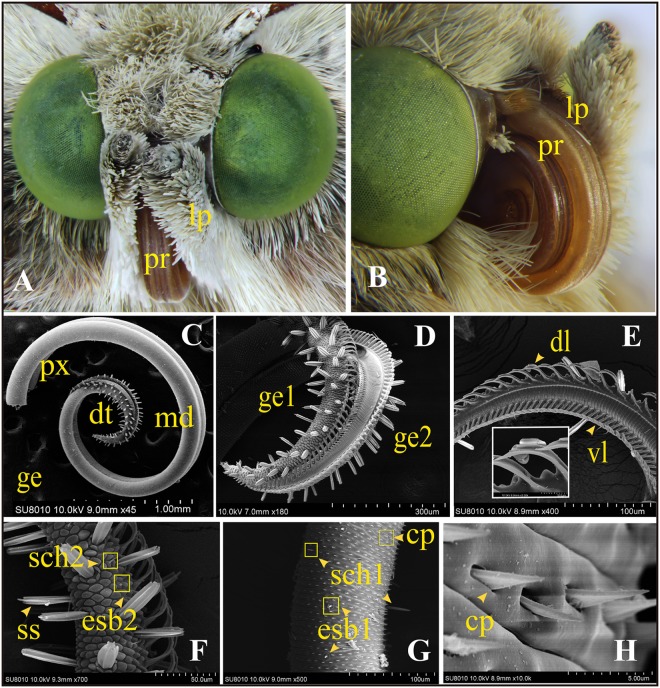
General morphology of the mouthpart organs and ultrastructure of the proboscis of adult *Helicoverpa armigera*. **(A)** Frontal view of the head shows the major siphoning mouthpart organs: proboscis (pr) and the pair of labial palps (lp). **(B)** The proboscis (pr) coiled up under resting state; one labial palp attached on the side (the other one was removed). **(C–H)** Scanning electron micrographs of proboscis. **(C)** Overall structure of proboscis: the rough distal section (dt) and the smooth proximal (px) and middle (md) sections were shown on the two elongated galeae (ge). **(D)** The distal section (dt) of two galeae shows many peg-shaped sensilla. **(E)** Dorsal (dl) and ventral ligulae (vl) on each galea. **(F)** Two major sensilla on the distal section: sensilla styloconica (ss), external sensilla basiconica subtype 2 (esb2) and sensilla chaetica subtype 2 (sch2). **(G)** Two types of sensilla on the proximal and middle sections: external sensilla basiconica subtype 1 (esb1) and sensilla chaetica subtype 1 (sch1). Plenty of cuticular processes (cp) arranged on the surface. **(H)** Triangular structure of cuticular processes.

Adults of *H. armigera* possess a typical siphoning mouthpart, which consists of two main organs: proboscis and labial palps (**Figure 1A**). The proboscis is coiled up completely and attached by a pair of labial palps on each side in the resting state (**Figure 1B**). When feeding or detecting, the proboscis is stretched out as a long tube and the labial palps are twisted around. The fine structure of the labial palps, which are prominent structures in the front of the head, has been studied by Zhao et al. (2013) in detail. The function of labial palps was considered to be closely related to its structure. We performed electron microscope scan on the proboscis to observe the morphology and structure.

#### The Overall Structure of the Proboscis

The proboscis is a tubular structure, which consists of the two elongated galeae (ge) (**Figures 1C,D**). The dorsal (dl) and ventral ligulae (vl) on each galea (**Figure 1E**) are joined together, which create the capillary construction for sucking liquids. The distal region (dt) was covered with abundant peg-shaped sensilla and appeared rough (**Figures 1C,D**). This area was equipped with all the three major types of sensilla: the two subtypes of sensilla styloconica (ss1 and ss2), one subtype of sensilla basiconica (esb2), and one subtype of sensilla chaetica (sch2) (**Figure 1F**). The proximal (px) and middle (md) sections of the proboscis possessed a smooth exocuticle, with numerous triangular cuticular processes (cp) (**Figure 1H**) together with two major types of sensilla: basiconica (esb1) and one sensilla chaetica (sch1) (**Figure 1G**).

#### Proboscis Sensilla in Adult *H. armigera*

A total of three major types of sensilla including nine subtypes were observed on the proboscis of male and female moths: sensilla styloconica (ss1 and ss2), sensilla chaetica (sch1 and sch2), and sensilla basiconica (esb1, esb2, esb3, isb1, and isb2).

##### Sensilla styloconica

**FIGURE 2:**
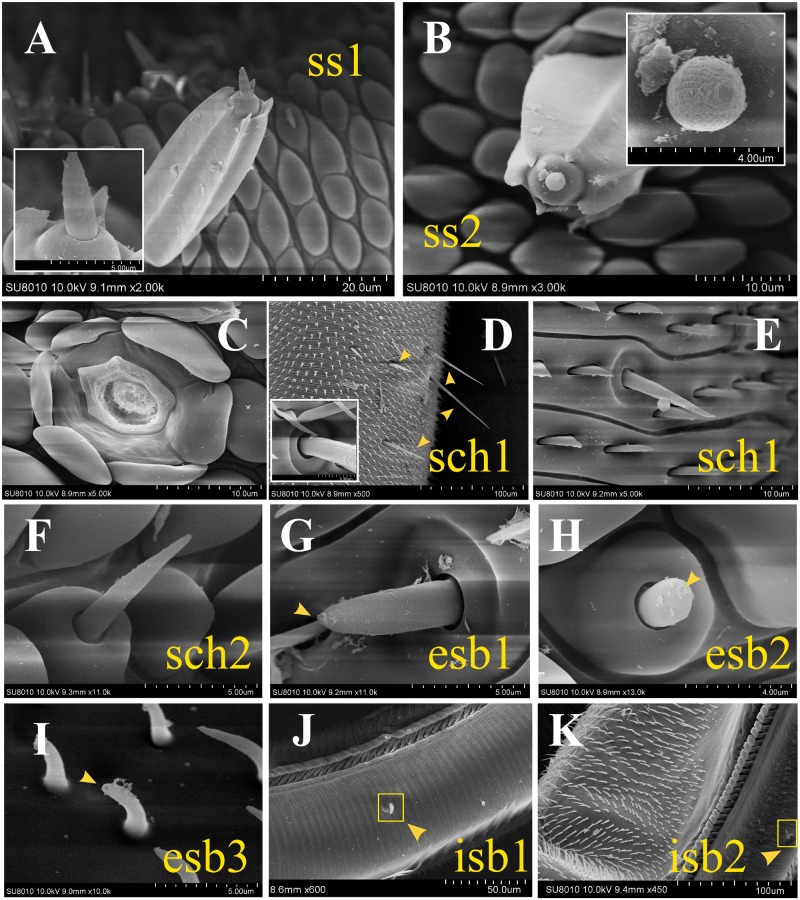
Scanning electron micrographs of sensilla on the proboscis of adult *H. armigera*. **(A)** Sensilla styloconica subtype 1 (ss1) possessing a uniporous cone. **(B)** Sensilla styloconica subtype 2 (ss2) with a multiparous sphere. **(C)** The lotus-shaped pedestal of sensilla styloconica. **(D)** Long and short sensilla chaetica subtype 1 (sch1) on the proximal section. The cupped socket at the base of sch1 (white box). **(E)** Short sensilla chaetica subtype 1 (sch1) on the middle section. **(F)** Sensillum chaetica type 2 (sch 2) on the distal part of the proboscis. **(G)** External sensilla basiconica subtype 1 (esb1) with a basal socket and a top pore. **(H)** External sensilla basiconica subtype 2 (esb2) on a roof-shaped bulge and with a pore on the tip. **(I)** External sensilla basiconica type 3 (esb3) with an uniporous peak and a curving cone. **(J)** Internal sensilla basiconica type 1 (isb1) on the internal surface of the proboscis tube. **(K)** Internal sensilla basiconica type 2 (isb2).

A large number of sensilla styloconica were present (about 60 on each galea) on the proboscis, and they were arranged only in the distal region and were nearly perpendicular to the cuticula of the proboscis. Each sensillum was composed of a large peg-shaped protrusion with a large cavity inside and six ridges outside on the longitudinal direction. A lotus-shaped pedestal was present at the base of each sensillum (**Figure 2C**). A roof-shaped bulge was observed above each peg. Two subtypes have been identified according to the composition of each bulge. Sensilla styloconica type 1 (ss1) has a uniporous smooth cone (**Figure 2A**), the top of which possesses a pore of about 0.2 μm in diameter. The largest number of ss1 was observed on the distal region. The other subtype of sensilla styloconica, ss2 (**Figure 2B**), has a sphere on the tip. The surface of a single sphere was covered by a longitudinal groove, containing numerous micropores. Ss2 was the only multiporous sensilla type what we found on the proboscis, and a low number were interspersed among the ss1.

##### Sensilla chaetica

Sensilla chaetica was a cuspidal bristle-shaped structure with longitudinal lines on the surface. Two subtypes of sensilla chaetica were classified according to the features at their base. Both of the two subtypes were uniporous on the top. The base of sensilla chaetica type 1 (sch1) was aporous (**Figures 2D,E**). Each of them was inserted into a cupped socket and was located only in the proximal and middle regions. The length of these sensilla varied greatly from about 10 to 70 μm. The longer sch1 only existed in the proximal part of the proboscis. The shorter sch1 was scattered in the proximal and middle sections. Sensillum chaetica type 2 (sch 2) (**Figure 2F**) inserted its base into a roof-shaped bulge and had a similar pyramid appearance with the shorter type 1. This subtype of sensilla was only located on the distal part of the proboscis.

##### Sensilla basiconica

Sensilla basiconica was typically composed of a blunt, short peg-shaped cone with a terminal pore. Three subtypes were found on the external surface of the proboscis. Each external sensilla basiconica type 1 (esb1) (**Figure 2G**) inserted its base into cupped sockets and were present only on the proximal and middle parts. External sensilla basiconica type 2 (esb2) (**Figure 2H**) was located on a roof-shaped bulge and was only present on the distal section. External sensilla basiconica type 3 (esb3) had a uniporous peak and a curving cone (**Figure 2I**). This subtype only existed in the ventral side of the proximal galeae and has not been described in any adult noctuidae. We named them sensilla basiconica because they are similar to some previously reported basiconica-type sensilla (Xue et al., 2016). Furthermore, the two subtypes of sensilla basiconica were identified on the internal surface of the proboscis; both were low in number. Internal sensilla basiconica type 1 (isb1) (**Figure 2J**) possessed a similar cone with esb3 but had a cylindrical depression at the base. The morphology of internal sensilla basiconica type 2 (isb2) (**Figure 2K**) was the same as esb1.

### Identification of Chemosensory Genes in the Mouthpart Organs

A great number of sensilla with various morphologies have been described in the mouthpart organs earlier. Subsequently, further research on chemosensory genes was conducted by transcriptomics.

#### Sequencing and Assembly

The mouthpart transcriptome of adult *H. armigera* was obtained through Illumina Hiseq2000. A total of 99,606,218 and 108,678,674 raw reads were obtained from the proboscis and labial palp transcriptomes, respectively. Then, 97,650,394 and 106,569,066 clean reads with a Q20 percentage of 98.45 and 98.38%, respectively, were assembled into 88,983 and 116,096 contigs, respectively, using Trinity assembler. Finally, 43,405 unigenes were assembled by combining the data of proboscis with labial palp. This dataset consists of 43,405 unigenes including 14,297 distinct clusters and 29,108 distinct singletons with a mean length of 1,256 nt and N50 of 2,578 nt. A blastx algorithm against the NCBI non-redundant protein database revealed that 23,563 unigenes shared sequence similarities with known proteins using (cutoff *E*-value of 10^-5^). Homology analysis with other insect species indicated that the dataset shared the best match with *B. mori* (26.5%), followed by *Danaus plexippus* (15.50%), and *Papilio xuthus* (1.46%).

#### Identification of Candidate Chemosensory Genes

##### Chemosensory receptors

**FIGURE 3:**
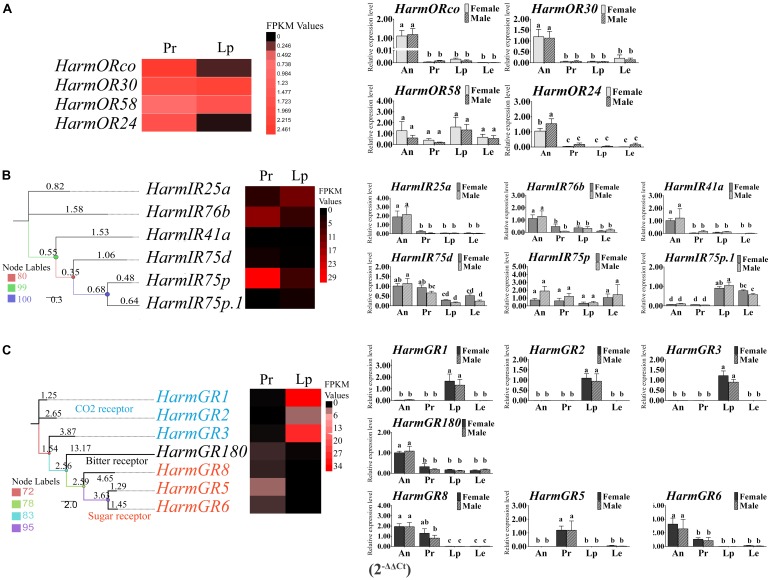
The heat map of the expression level of ORs, IRs, and GRs based on the DEGs analysis and the relative expression level based on the qRT-PCR. The heat maps were generate based on the FPKM values. The column diagrams representing the relative expression level of each gene between four tissues of both sexes were generated based on 2^-ΔΔCt^. The differences of expression among tissues and sexes were analyzed by one-way ANOVA and followed by Duncan’s test (*P* < 0.05). Different letters on the top of the columns represent significant difference at *P* < 0.05. **(A)** Expression level of ORs; **(B)** expression level of IRs; **(C)** expression level of GRs; An, antenna; Pr, proboscis; Lp, labial palps; Le, legs.

Four candidate ORs, based on a series of strategies, were identified through transcriptome analysis. All of these genes turned out to be previously reported ORs by Blast homology analysis. The reported co-receptor *HarmOrco*, performing function by co-expressing with specific OR, was identified with a complete open reading frame (ORF). Partial sequences of *HarmOR24*, *HarmOR30*, and *HarmOR58* were obtained (**Supplementary Table S1**). *HarmOR58*, which was detected only in larval antenna by previous reports, was also found here. A total of six transcripts of candidate IRs were identified in the mouthparts. Blast homology analysis indicated that they belong to the previously reported 21 IRs. Complete ORFs of three IRs (*HarmIR25a, 76b*, and *41a*) were obtained, and the sequences of the other three IRs (*HarmIR75d, IR75p*, and *IR75p.1*) were partial (**Supplementary Table S1**). Seven candidate GRs were screened in our dataset including four long sequences, two of which had complete ORFs. All of them were identified as the known GRs with identities from 98 to 100% according to the Blastx homology analysis (**Supplementary Table S1**). A phylogenetic tree of the seven GRs was generated (**Figure 3C**). *HarmGR1-GR3* belonged to the reported CO_2_ receptor clade; *HarmGR5*, *GR6*, and *GR8* were part of the “sugar” receptor group; and *HarmGR180* was part of the “bitter” receptor subfamily, which was suggested to be the most extended subfamily. The transcript levels of each receptor gene were initially estimated based on the FPKM values. *HarmORco* was expressed in the proboscis with the highest level followed by OR30, OR24, and OR58. In the labial palp, unexpectedly, HarmOR30 and OR58 had the most abundant expression (**Figure 3**: A-heat map). The heat map of the six IRs revealed that *HarmIR75p* had the highest expression level in the proboscis followed by *HarmIR76b*. In the labial palps, *HarmIR25a* was expressed at a higher level than the other five IRs (**Figure 3**: B-heat map). For the GRs, their expression in proboscis and labial palps exhibited three patterns. *HarmGR1, GR2*, and *GR3* were mainly expressed in labial palps, whereas *HarmGR5, GR5*, and *GR8* were mainly expressed in proboscis. *HarmGR180* has similar expression in both two tissues (**Figure 3**: C-heat map).

To confirm the DEG results of the three families of receptor genes, we performed qRT-PCR in four major tissues including the antenna, proboscis, labial palps, and legs of both sexes. All the ORs were detected in all the four tissues. Most of the ORs were expressed in the antenna with significant higher level than the other tissues (*P* < 0.05) except *HarmOR58*, which was expressed in labial palps with a greater abundance than in other tissues but no significant difference (*P* > 0.05) (**Figure 3**: A-histogram). For the IRs, the expression of *HarmIR25a, 76b. 41a* in the antenna was significantly higher than that in others tissues (*P* < 0.05). The expression patterns of *HarmIR75d, 75p, 75p.1* turned out to be diverse. *HarmIR75d* was mainly expressed in the antenna and proboscis; *HarmIR75p* was expressed in all the tissues with no significant difference; in particular, *HarmIR75p.1* was mainly expressed in the labial palps and legs (**Figure 3**: B-histogram). Most of GRs were abundantly expressed in proboscis or labial palps. The expression of *HarmGR1, GR2*, and *GR3* in labial palps was significantly higher than that of other tissues (*P* < 0.05), and that of *HarmGR5* in proboscis was significantly higher than other tissues (*P* < 0.05). *HarmGR6* and *GR8* were mainly expressed in antenna and proboscis. *HarmGR180* was mainly expressed in the antenna, and its expression level in other tissues was similar to each other (**Figure 3**: C-histogram).

##### Abundant expression of soluble proteins

**FIGURE 4:**
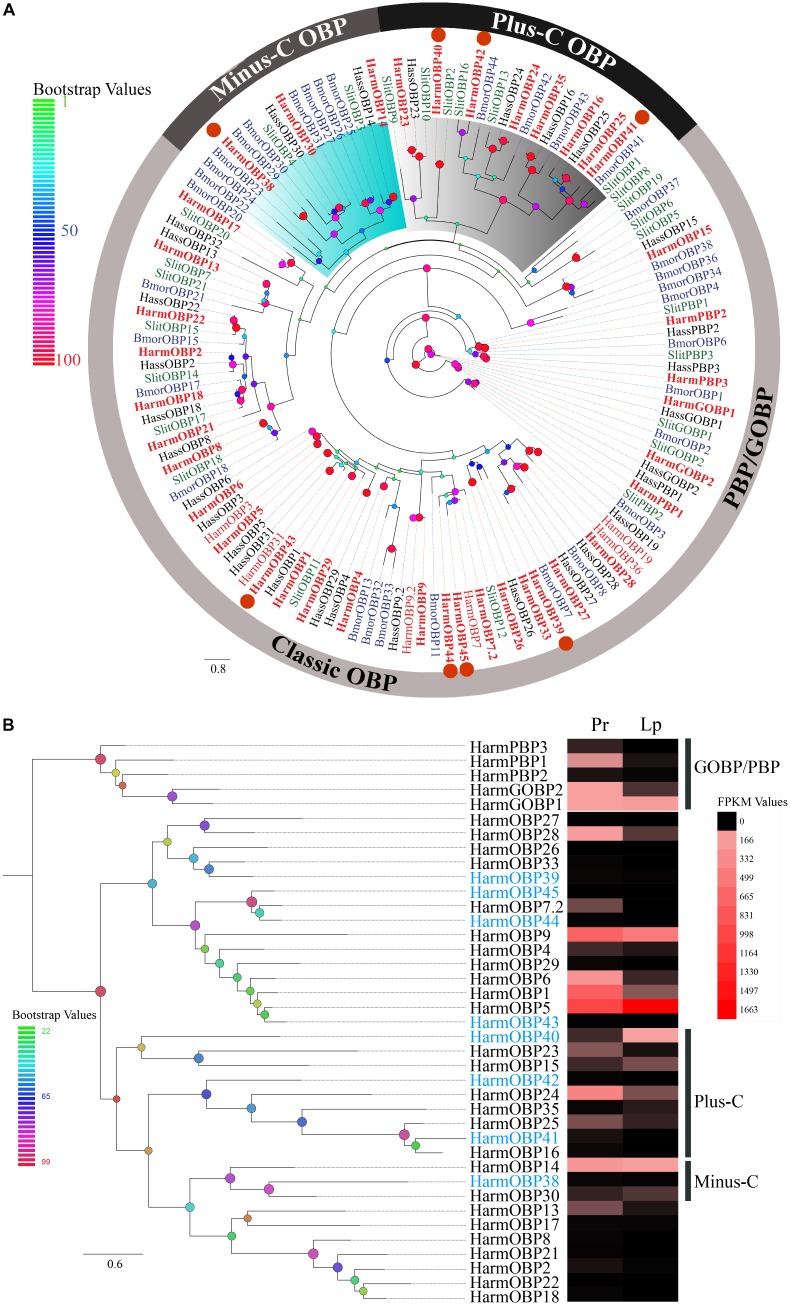
**(A)** The phylogenetic tree of OBPs from Lepidoptera species. The OBPs identified in our dataset are identified in bolding font. Eight novel OBPs are marked by orange circles. The “classic” OBP clade including PBP/GOBP, “plus-C” OBP, and “minus-C” is shown. Bootstrap values are shown by color gradation. The four species re *H. armigera* (Harm, red), *H. assulta* (Hass, black), *S. littoralis* (Slit, green), and *B. mori* (Bmor, blue). **(B)** Phylogenetic tree of 39 OBPs identified in our dataset. Their expression profiles were shown by a heat map based on the FPKM values. Eight novel OBPs are marked in blue font.

We identified 39 OBP and 26 CSP transcripts of two small soluble protein groups. Eight novel OBPs were found, together with 31 previously reported genes (**Supplementary Table S1**). A total of 26 of 39 OBPs were identified as full-length sequences with complete ORFs and 34 amino acid sequences with signal peptides. A phylogenetic tree was constructed using 134 OBPs from four Lepidoptera species including the 39 transcripts identified in mouthpart organs. These OBPs were generally clustered into three subfamilies (**Figure 4A**). The “classic” OBP group contained the most members including general odorant-binding protein (GOBP) and pheromone-binding protein (PBP) with six conserved cysteines. Members of the “minus-C” group had only four cysteines, whereas the “plus-C” group had more than six cysteines (Zhou et al., 2004; Gong et al., 2009; Gu et al., 2015; Chang et al., 2017; Wang et al., 2017). The four novel OBPs (*HarmOBP39, 43, 44*, and *45*) together with 22 reported OBPs were part of the “classic” OBP group, *HarmOBP38* belonged to the “minus C,” and the three novel OBPs (*HarmOBP40, 41*, and *42*) belonged to the “plus C” groups. Sequence alignment (**Supplementary Figure S1A**) showed the same pattern as the phylogenetic tree except for *HarmOBP9*, which was clustered into the “classic” OBP clade, although it has only five conserved cysteines. Transcript levels of the identified OBPs were initially estimated based on the FPKM values. The majority of OBPs were expressed in the proboscis or labial palps at a high level (**Figure 4B**). More OBPs including *HarmOBP5, OBP9, OBP1*, and *OBP24* were found in the proboscis with a higher expression level than in labial palps. *HarmOBP5* had the highest FPKM value in labial palps, followed by *HarmOBP9*. The expression level of the eight novel genes was lower except for *HarmOBP40*.

**FIGURE 5:**
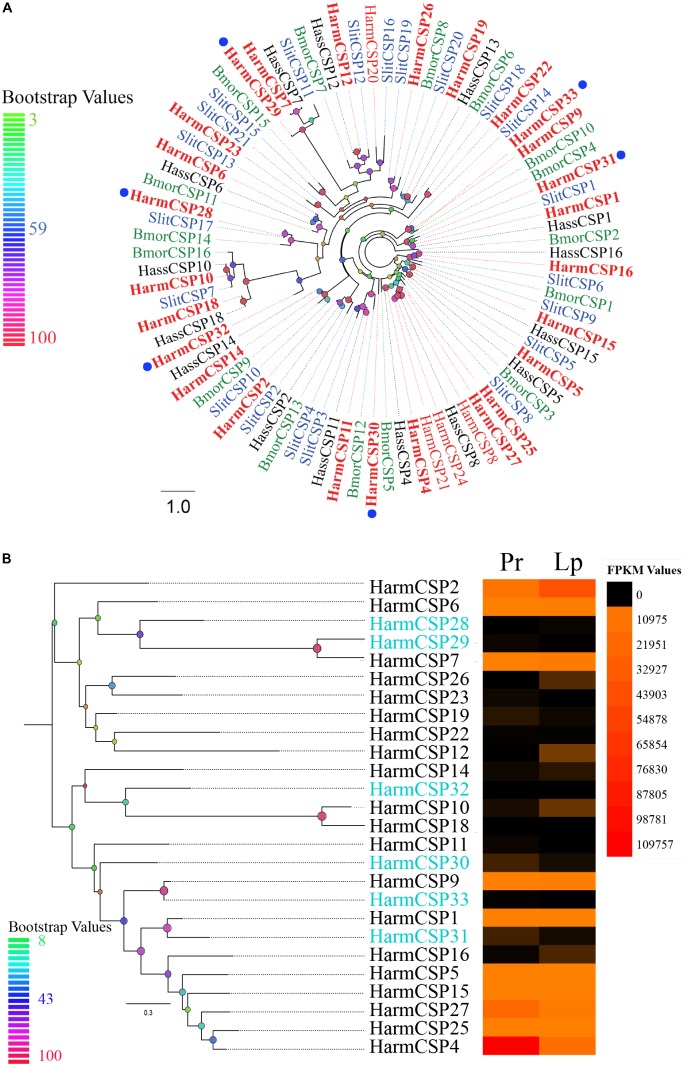
**(A)** The phylogenetic tree of CSPs from Lepidoptera species. The CSPs identified in our dataset are shown in bolding font. Six novel CSPs are marked by blue circles. Bootstrap values are shown by color gradation. The four species are *H. armigera* (Harm, red), *H. assulta* (Hass, black), *S. littoralis* (Slit, blue), and *B. mori* (Bmor, green). **(B)** Phylogenetic tree of 26 CSPs identified in our dataset. Their expression profiles were shown by a heat map based on the FPKM values. Six novel CSPs are marked in blue font.

Six novel CSPs with the addition of 20 reported genes were identified in the transcriptome of the proboscis and labial palps. A total of 20 of 26 CSPs had complete ORFs. Further analysis showed that 24 CSPs covering the six novel genes had signal peptides on the N-terminal end of their amino acid sequences (**Supplementary Table S1**). A phylogenetic tree of 82 CSPs in *H. armigera*, *H. assulta*, *Spodoptera littoralis*, and *B. mori* was inferred to investigate the homology among sequences. It was shown that the six novel CSPs in our study were orthologous with those in other species (**Figure 5A**). Sequence alignment suggested that all 26 CSPs contained four conserved cysteine residues except *HarmCSP25*, for which the ORF was partial (**Supplementary Figure S1B**). The expression level of each CSP was visualized by the heat map based on the FPKM values (**Figure 5B**). The expression level of several CSPs (*HarmCSP4*, *27*, *2*, *6*, *7*, *9*, *1*, *5*, *15*, and *25*) was extremely high in both proboscis and labial palps. The expression levels of all six novel genes were lower. *HarmCSP4* was expressed in the proboscis at an especially high level, and the FPKM value was 109,757. In labial palps, the most abundantly expressed gene was *HarmCSP2*.

##### Sensory neuron membrane proteins

**FIGURE 6:**
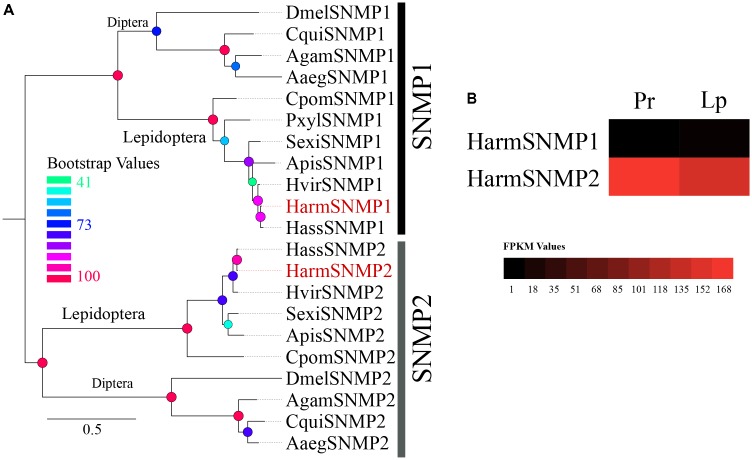
**(A)** The phylogenetic tree of 21 reported SNMPs in 11 species revealed two separate clades of SNMP1 and SNMP2. **(B)** The expression profiles of two SNMPs identified in proboscis and labial palp were shown by a heat map based on the FPKM values.

The two reported SNMPs (SNMP1 and SNMP2), which were first identified in the antenna, were identified in our dataset with complete ORFs (**Supplementary Table S1**). A phylogenetic tree of 21 reported SNMPS in 11 species revealed two separated clades of SNMP1 and SNMP2 (**Figure 6A**). The transcript level of the two SNMPs based on the FPKM values in different tissues suggested that the expression level of SNMP2 was very high in both proboscis and labial palps, whereas the expression level of SNMP1 was very low (**Figure 6B**).

## Discussion

In the last few decades, many studies on the host recognition of insects have been performed using molecular biology methods, and much attention has been focused on the antenna, which is regarded as the primary olfactory organ. The mouthparts, however, also play crucial roles in biological activity such as finding host plants or feeding. The general morphology of the proboscis in *H. armigera* has been described in previous studies (Blaney and Simmonds, 1988; Wang et al., 2012). Here, we investigated the fine structure of the proboscis of adult *H. armigera* in detail. A total of nine subtypes belonging to the three major types of sensilla (sensilla styloconica, sensilla basiconica, and sensilla chaetica) were identified on the proboscis, and three subtypes (ss2, esb3, and isb1) were identified for the first time.

The most abundant sensilla were found at the terminal section, where fluid can be sucked up. Two subtypes of sensilla styloconica were identified according to the characters of their tips. Subtype 1 (ss1) possessed a cuspidal cone on top with a terminal uniporous. This type of sensilla was considered to be one of the most common types among most lepidopterans (Faucheux, 2013). In three noctuidae moths including *H. armigera*, the function of this type was previously identified as contact chemoreception by Blaney and Simmonds (1988). The sensilla responded to several substances such as “sugars” (glucose, fructose, sucrose, and others), nicotine, and amino acids (gamma-aminobutyric acid) (Blaney and Simmonds, 1988). The subtype 2 (ss2) was multiporous on the wall of the tip sphere. They were located among the top uniporous subtype 1 at a low number. This type of sensilla was first found in *H. armigera* and was rarely described in other noctuidae species, which could be due to the small number of sensilla or the resolution ratio of images. The subtype 2 was similar to the uniporous-multiporous sensilla styloconica (UP-MP ss) and possessed a terminal pore and wall pores at the terminal structure probably as the combination of gustatory and olfactory sensilla (Faucheux, 2013). The subtype 2 on the proboscis of *H. armigera* was wall-multiporous but without the top uni-pore. We theorized that this type of sensilla functioned as olfactory chemoreceptors, which may sense plant volatiles before finally sucking food. Sensilla chaetica subtype 1 (sch1) was also described by a previous study but was wrongly classified as “trichodea” due to their long hair-like outlines (Wang et al., 2012). The typical characteristic of sensilla chaetica in most Lepidoptera, however, is the longitudinal ridge surface and the basal socket (Faucheux, 2013; Xue and Hua, 2014). Sensilla basiconica esb3 and isb1 were described for the first time in *H. armigera*.

After identifying various sensilla types on the proboscis and labial palps, which suggest a comprehensive chemosensory system in the mouthpart organs of *H. armigera*, we then systematically mined the candidate chemosensory genes in the proboscis and labial palps. We obtained data of 4 ORs, 6 IRs, 7 GRs, 39 OBPs, 26 CSPs, and 2 SNMPs. We rarely detected ORs in the mouthpart transcriptome of adult *H. armigera*. Four of the 66 reported ORs were identified. *HarmOrco*, which was considered an atypical co-receptor, had the highest expression level in the proboscis and labial palps. *HarmOR58*, which has been identified as a larval antennal specific gene by previous work (Liu N.Y. et al., 2014), was also detected in all the four tissues but with the low expression level. These ORs in mouthpart organs might play roles beyond food finding.

As another class of chemosensory receptor, IRs were suggested to mediate detection of certain chemical stimuli, predominantly to acids, aldehydes, and amines (Benton et al., 2009). Studies on *Drosophila melanogaster* revealed that *IR64a* is co-expressed with *IR8a* to form a functional ligand-gated ion channel for acid sensing *in vivo* (Ai et al., 2013). *IR84a*-expressing neurons in *D. melanogaster* were activated by phenylacetic acid and phenylacetaldehyde, which were regarded as the signal of food sources and oviposition sites and contributed to courtship (Grosjean et al., 2011). Apart from olfaction sensing, some IRs were suggested to play versatile roles in taste perception (salt, amino acids, etc.) and temperature sensing (Rimal and Lee, 2018). Six IRs were identified in the mouthparts based on our dataset. *HarmIR25a*, which belongs to the most conserved clade of the IR family among species and acts as a co-receptor (Croset et al., 2010), exhibited the most abundant expression in labial palps. In contrast, research on *Drosophila* suggested that *IR25a* was involved in temperature sensing in the chordotonal organ (Chen et al., 2015). It can be speculated that *HarmIR25a* might play roles in temperature perception as the highly conserved properties of IR sequences among species. Analogously, *HarmIR76b* might be the receptor for sensing amino acids or salt based on the studies in *Drosophila* (Zhang et al., 2013; Ganguly et al., 2017). *HarmIR75p* exhibited the most abundant expression in proboscis based on the DEG analysis; however, the qRT-PCR results suggested the lower expression level than *HarmIR76b* and *IR25a.*

Within the GR family, there are two well-studied subsets: CO_2_ receptors and “sugar” receptors. Seven GRs were identified in the mouthparts. *HarmGR1, GR2*, and *GR3* have been reported as CO_2_ receptors that are mainly expressed in labial palps. *HarmGR1* and *HarmGR3* have been reported to respond robustly to NaHCO_3_ when they are co-expressed (Ning et al., 2016). *HarmGR5, GR6*, and *GR8*, which were mainly expressed in the proboscis, were part of the “sugar” GR clade. As mentioned earlier, electrophysiological experiments on the proboscis of *H. armigera* have demonstrated that many sensilla styloconica subtype 1 (ss1) respond to sugars, nicotine, and some amino acids. Previous studies have identified HarmGR4 and HarmGR9 as the receptors of several sugars (Xu et al., 2012; Jiang et al., 2015). These two GRs were not identified in the mouthpart organs based on our dataset, but *HarmGR5, GR6*, and *GR8* belong to the same clade as *HarmGR4*. It could be that one of the three GRs we found, or their combination, is used for sensing sugar. *HarmGR180* was part of the “bitter” receptor subfamily, which is the largest clade in the GR family (Xu et al., 2016). The only “bitter” GR might be the receptor of some alkaloids such as nicotine or some amino acids. Further, these sugar and bitter GRs are probably expressed in styloconica subtype 1.

We sequenced many small soluble proteins (39 OBPs and 26 CSPs), among which eight OBPs and six CSPs were identified for the first time. After the first OBP and CSP were discovered in the giant moth *Antheraea polyphemus* and *D. melanogaster*, respectively (Vogt and Riddiford, 1981; Mckenna et al., 1994), a large number of OBPs and CSPs have been identified in many insects. Certain OBPs and CSPs have been reported to move volatile molecules (Zhang et al., 2012; Li et al., 2013) to the membrane of chemosensory neurons, where transmembrane receptors (ORs, GRs, or IRs) are expressed. However, the reason for the large number of OBPs and CSPs in the mouthpart organs where a minority of receptor genes were found is unknown. The most likely explanation is that non-sensory functions were endowed to certain OBPs and CSPs beyond chemo-signal detection. It has been reported that OBP22 of mosquito *Aedes aegypti* was produced in the sperm and transferred to females (Li et al., 2008). Certain OBPs/CSPs were described in many activities including development, anti-inflammation, carrying visual pigments, insecticide resistance, and so on (see review of Pelosi et al., 2017).

The two subfamilies of insect SNMPs (SNMP1 and SNMP2), two transmembrane domain receptor proteins homologous to the mammalian CD36 receptor (a family of proteins whose members frequently interact with proteinaceous ligands) (Rogers et al., 2001; Jiang et al., 2016), were identified in the dataset with complete ORFs. Studies have shown that the SNMP1 subtype is co-expressed with PRs in pheromone sensory neurons and contributes to the sensitivity of pheromone sensing in several insect species. In contrast, SNMP2 was localized in the supporting cells of neurons (Benton et al., 2007; Forstner et al., 2008; Jin et al., 2008; Liu C. et al., 2014; Pregitzer et al., 2014; Jiang et al., 2016). The function of SNMP2 has not yet been identified. Based on our data, the transcript level was very high in both proboscis and labial palps, which suggests a role of SNMP beyond pheromone sensing.

In summary, these results suggest that the mouthparts are a mutually cooperative but functionally concentrated system. Our results contribute to the understanding of chemical signal recognition in mouthpart organs. Further functional studies about certain chemosensory proteins such as receptors, which were identified in proboscis and labial palps, need to be conducted. On one hand, these would help to investigate the physiological activities of moths when they are feeding. On the other hand, more target genes could be used in the pest management.

## Author Contributions

GW and ZH conceived the study. GW and YL acquired the grant, also participated in its design, coordination, and supervision. MG carried out the laboratory experiments with contributions from QC. MG analyzed the data and wrote the paper. All authors read the paper and gave final approval for publication.

## Conflict of Interest Statement

The authors declare that the research was conducted in the absence of any commercial or financial relationships that could be construed as a potential conflict of interest.

## Footnotes

1 http://web.expasy.org/translate/

2 http://www.cbs.dtu.dk/services/SignalP/

3 https://www.ebi.ac.uk/Tools/msa/mafft/

4 http://itol.embl.de/

## Supplementary Material

FIGURE S1 Alignments of amino acid sequences of OBPs and CSPs identified in the proboscis and labial palps of *H. armigera*. **(A)** Conserved cysteines of *HarmOBPs* were shown by C1–C6. Eight novel OBPs were marked by orange circles. **(B)** Conserved cysteines of *HarmCSPs* were shown by C1–C4. Six novel CSPs were marked by orange circles.

TABLE S1 Sequence analysis of candidate chemosensory genes (4 ORs, 6 IRs, 7 GRs, 39 OBPs, 26 CSPs, 2 SNMPs) identified in the proboscis and labial palps of *H. armigera*.

TABLE S2 Primers used in qRT-PCR.

FILE S1 Unigenes of eight novel odorant binding proteins (OBPs) and six novel chemosensory proteins (CSPs) identified in the proboscis and labial palp.
