# Supplementary figures and images for: Chemoreception of Mouthparts: Sensilla Morphology and Discovery of Chemosensory Genes in Proboscis and Labial Palps of Adult Helicoverpa armigera (Lepidoptera: Noctuidae)
Source: Front Physiol. 2018 Aug 7;9:970. doi: 10.3389/fphys.2018.00970 (PMC6091246; doi:10.3389/fphys.2018.00970)

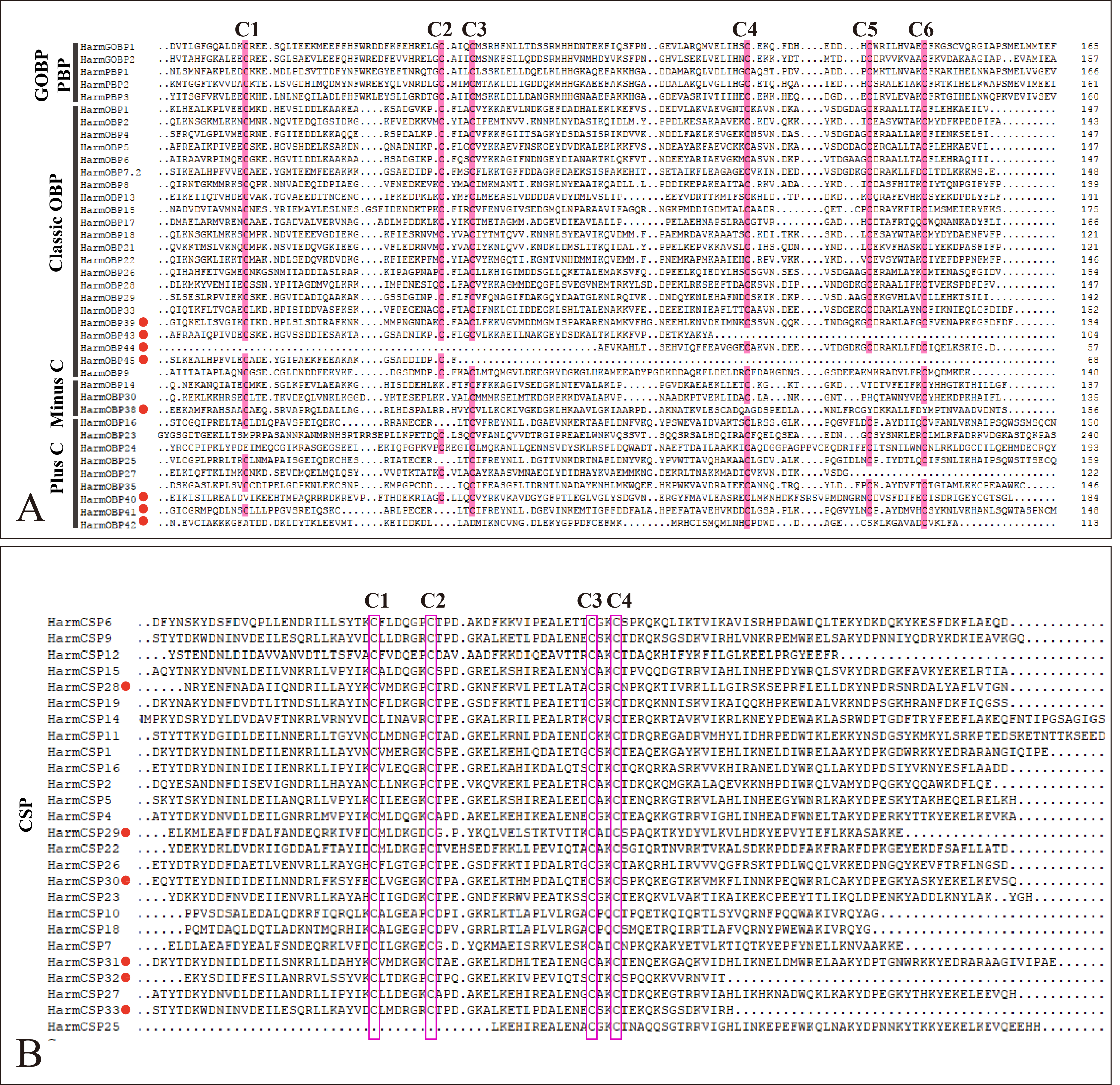

Supplement: FIGURE S1 — Alignments of amino acid sequences of OBPs and CSPs identified in the proboscis and labial palps of H. armigera. (A) Conserved cysteines of HarmOBPs were shown by C1–C6. Eight novel OBPs were marked by orange circles. (B) Conserved cysteines of HarmCSPs were shown by C1–C4. Six novel CSPs were marked by orange circles. [file Image_1.TIF]
