# Supplementary material for: Chemoreception of Mouthparts: Sensilla Morphology and Discovery of Chemosensory Genes in Proboscis and Labial Palps of Adult Helicoverpa armigera (Lepidoptera: Noctuidae)
Source: Front Physiol. 2018 Aug 7;9:970. doi: 10.3389/fphys.2018.00970 (PMC6091246; doi:10.3389/fphys.2018.00970)
Supplement: FILE S1 — Unigenes of eight novel odorant binding proteins (OBPs) and six novel chemosensory proteins (CSPs) identified in the proboscis and labial palp. [file Data_Sheet_1.DOCX]

**File S1_**Unigenes of eight novel OBPs and six novel CSPs identified in the proboscis and labial palp.

**OBPs**

>Unigene5174_All-*HarmOBP38*

ATGACTTGCTCTCGTGCGCTGGCGCTGCTCGCGCTCGTCGCTCTCACGCAACAGGCAACAACAGTATGCAAGAATTGTATAATGCTGGGTAAGGAGGAGAAGGCGATGTTCCGCGCGCACTCGGCGGCGTGCGCGGAGCAGTCGCGCGTGGCGCCGCGGCAGCTGGACGCGCTGCTGGCGGGCCGCCTGCACGACTCGCCCGCGCTGCGCCGCCACGTCTACTGCGTGCTGCTCAAGTGCAAGCTGGTGGGCAAGGACGGCAAGCTGCACAAGGCCGCCGTGCTCGGCAAGATCGCCGCCAGGCCTGATGCTAAGAACGCTACCAAGGTCCTGGAGAGCTGCGCAGACCAGGCGGGCGACTCGCCCGAGGACCTGGCGTGGAACCTGTTCCGCTGCGGCTACGACAAGAAGGCGCTGCTCTTCGACTACATGCCCACCAACGTGGCGGCCGACGTCGACAACACCTCCTAG

>Unigene6393_All-*HarmOBP39*

GCAATTTCGGACGCGCAGAAATCTGGCATACAAAAAGAGTTAATAAGCGTTGGTATAAAATGCATTAAGGATCATCCCCTATCACTATCTGATATAAGGGCGTTTAAAAACAAAATGATGCCCAATGGGAACGATGCTAAATGTTTCGCTGCGTGTTTGTTCAAGAAGGTTGGAGTGATGGACGATATGGGAATGATATCACCAGCTAAAGCACGAGAAAACGCCATGAAGGTGTTCCATGGGAATGAAGAACATTTGAAGAACGTTGATGAAATTATGAATAAATGTTCCTCAGTGAATCAACAAAAAACAAATGACGGACAAAAAGGCTGTGATAGAGCAAAGCTAGCATTTGGATGTTTTGTAGAGAATGCTCCTAAATTCGGATTCGATTTTGATTTCTGA

>Unigene5118_All-*HarmOBP40*

ATGGTTCGCAAAATCGGTGGACTTTTGTGTTGTCTTTGCGTTTTCGGCATTTCGCTGAGTGATAGCGCCATATCAGCTGATTCTGAGTCACGATGCCGCAACCCGCCTACGGCTCCTCAGAAGATAGAACGAGTCATAACTCTGTGTCAGGATGAAATCAAACTGTCCATACTAAGAGAGGCTCTTGACGTGATCAAAGAAGAACACACGATGCCTGCACAGAGGCGGCGCGACAAGCGAGAAGTGCCCTTCACGCACGACGAGAAGAGAATTGCTGGGTGCTTGCTGCAGTGTGTGTACAGAAAAGTGAAAGCCGTGGACGGCTACGGTTTCCCGACGCTGGAAGGTCTGGTGGGTCTGTACTCGGACGGCGTGAACGAGCGCGGCTACTTCATGGCGGTGCTCGAGGCCTCGCGCGAGTGCCTCATGAAGAACCACGATAAATTCTCTAGATCTGTTCCTATGGACAACGGACGCAACTGCGACGTTTCGTTCGACATTTTCGAGTGCATCTCTGACCGCATCGGCGAGTACTGCGGCACCTCGGGACTTTAA

>CL2042.Contig1_All-*HarmOBP41*

ATGTTTAAACTGTGCGTCTTCCTCGTGTTGTCTGTTGCGTCATGTTACGGCGGCGCCTCGGACAACGGCATTTGTGGGCGAATGCCCCAAGATCTAAATAGCTGTCTACTCCTCCCACCGGGTGTTAGTCGCGAGATTCAATCGAAATGTGCGAGATTACCAGAGTGCGAAAGACTGACCTGCATATTCCGTGAATACAACCTGCTGGACGGTGAAGTTATCAACAAGGAGATGACAATAGGATTCTTCGATGATTTTGCGCTGGCGCACCCTGAGTTTGCAACGGCTGTAGAACATGTGAAGGATGATTGCCTCGGCAGCGCTCCTCTCAAACCACAAGGAGTATACCTCAACTGTCCTGCCTATGATATGGTGCATTGTTCTTACAAGAATTTGGTGAAGCACGCGAATCTATCCCAGTGGACTGCTTCGCCGAACTGCATGCGCGCACGCACATTCGCCTCTTTGTGCCCCATCTGTCCTGACGAATGCTTCTCGCCAGAAGTGCCCAAAGGATCCTGTAATTGCTAG

>Unigene18374_All-*HarmOBP42*

AAGGAATGCTTCCAAATGCGTGGAAACCCAGTTACATGCGAAAACGAAGTATGTATAGCCAAGAAAAAAGGTTTTGCCACTGATGACGATAAATTGGACTACACAAAATTAGAAGAAGTGATGACCAAGGAAATTGATGACAAAGACCTTTTGGCAGACATGATAAAAAATTGTGTCAACGGAGATCTGGAGAAGTATGGGCCTCCTGACTTCTGTGAATTTATGAAGATGAGGCACTGTATCAGCATGCAAATGTTGAACCATTGTCCTGATTGGGATGACGCCGGTGAATGCAGCAAACTTAAAGGAGCAGTAGCAGACTGTGTGAAATTATTTGCGTGA

>Unigene19165_All-*HarmOBP43*

TTCACTTGTTTGGTTTTGTGTGTAGTGGCTGTTAGCATTAGCAAGGCTTATGCCAGTGAAGAAGACAAAGCAGCTTTCCGGGCAGCCATCCAGCCCATCGTAGACGAATGCTCGAAGGAGCATGGAGTCAGCTCTGACGATATCGAATCGGCCAAGACCGCTGGCAGCGCTGACAACATCAAGCCTTGCTTCCTCGGCTGCGTCTTGAAGAAAGCTGAAATTCTGAACGCCAAGGGAGAATATGATTCTGACAAAGCTCTGACTAAATTGAAGAAGTTCGTGCCCGATGAAACTAAATACGCTAAATACGCC

>Unigene18580_All-*HarmOBP44*

GCATTTGTCAAGGCCCATTTAACAAGCGAGCACGTCATCCAGTTCTTTGAAGCTGTTGGAGGAGAATGTGCCAAAGTGAATGACGAGGAAGTGACTGACGGTGATAAGGGGTGCGACAGAGCAAAGCTGTTGTTTGATTGTATACAGGAGCTCAAGTCAAAAATTGGCGATTGA

>Unigene17610_All-*HarmOBP45*

ATGTTTGGTTTCGGGTTCCTATCACTTGGGGCTGTGCTGCTTTGCTTGGGAAGCGCTATTGCTTTAACCCCAGAAGAAGAGTCAAGTCTGAAAGAGGCTTTGCATCCTTTTGTACTTGAATGTGCGGATGAATATGGAATACCTGCAGAGAAATTCGAGGAAGCGAAAGCCAAAGGCAGTGCTGACGATATTGATCCTTGTTTCAT

**CSPs**

>CL2988.Contig1_All-*HarmCSP28*

ATGAGGAGCTGGCTAATCTGCTTGTGCGTGCTGACGGTGGTGGTGACATGCCACTCGCAGGCCCCCAATCGCTATGAGAACTTCAACGCAGACGCCATCATTCAGAACGACAGGATCCTCCTGGCGTACTACAAGTGCGTCATGGATAAAGGACCCTGCACGAGGGATGGGAAGAACTTTAAACGTGTACTCCCAGAAACATTAGCCACCGCGTGCGGCCGCTGCAACCCCAAACAGAAGACGATCGTGCGCAAGCTACTCCTCGGCATCAGGTCCAAGAGCGAGCCTCGCTTCCTTGAGCTTCTTGACAAGTACAACCCTGATCGCTCCAACAGAGATGCCCTTTACGCTTTCTTAGTCACCGGCAACTAA

>Unigene11051_All-*HarmCSP29*

ATGAAAGTACTTATTGTGCTCACAGCGCTGGTTGCCTTCGCGGCTGCAGCGGCATTGACACCGGAAGAGCTAAAAATGCTTGAAGCTTTCGACTTCGACGCCTTGTTTGCCAATGATGAGCAGAGAAAGATAGTGTTCGATTGTATGCTGGACAAAGGGGATTGTGGTCCCTACAAACAGTTAGTAGAACTATCAACGAAGACTGTGACAACTAAATGTGCTGACTGCTCTCCTGCGCAGAAGACGAAGTACGATTATGTTCTGAAGGTGTTACACGACAAATATGAGCCTGTTTATACAGAGTTCCTGAAGAAGGCCAGCGCTAAAAAGGAATGA

>Unigene1671_All-*HarmCSP30*

ATGAAAACCTTATTCATTCTGTGTGCCCTCGTGATCGCCGTCAGCGCTCGTCCAGAAGAGCAGTACACAACCGAATATGATAATATAGACATTGATGAAATCTTAAATAATGACAGATTGTTCAAGAGCTATTTTGAATGCTTAGTAGGCGAGGGCAAGTGCACACCCGCAGGCAAGGAACTGAAAACTCATATGCCTGATGCACTACAGACCGAGTGCTCCAAGTGCTCGCCTAAGCAGAAAGAGGGAACCAAAAAGGTCATGAAATTCTTGATCAACAACAAGCCCGAGCAATGGAAGCGACTCTGCGCCAAATACGACCCCGAAGGCAAATACGCAAGCAAATATGAAAAAGAACTCAAAGAAGTCTCTCAATAA

>Unigene7342_All-*HarmCSP31*

ATGAAGTTCGTGCTACTGCTGTGCGTGATGGTCGCGGCTGTCGTCGCCGACGATAAGTACACTGACAAGTATGACAACATCGATCTGGATGAGATCCTCTCCAACAAGCGTCTTCTGGACGCCCATTACAAGTGTGTTATGGACAAGGGAAAGTGCACTGCTGAGGGCAAGGAGCTTAAAGACCACTTGACGGAAGCCATCGAGAACGGCTGCGCGAAATGCACGGAGAATCAAGAGAAGGGAGCACAGAAGGTTATCGACCACTTGATCAAGAATGAGCTGGATATGTGGCGTGAGCTGGCCGCCAAGTACGACCCTACTGGCAACTGGAGGAAGAAGTACGAGGACCGCGCGAGGGCTGCTGGCATCGTCATCCCCGCAGAATAA

>Unigene893_All-*HarmCSP32*

AAGTTCATTGTCTTCCCTCTCATATTCTGCGTTCTCCAGGTATGGTCGGCCCCAGCCGATGAAAAATATTCGGACATCGATTTCGAATCTATCCTAGCCAACAGGAGAGTGCTGTCTTCCTACGTCAAATGCCTCACCGATAAGGGACCATGCACACCTCAAGGAAAAGAACTGAAGAAAATCGTGCCAGAAGTGATTCAGACATCGTGCACAAAATGCAGTCCCCAACAGAAGAAGGTGGTGCGCAATGTGATCACC

>Unigene931_All-*HarmCSP33*

ATGAAGTTAATAGTTGCAGTTGCTTTACTATGCGTGGTGGCGATGGCCTGGGGTAAGCCCGCATCTACCTACACCGACAAATGGGACAACATCAACGTAGACGAGATCCTGGAGTCACAGCGCCTTCTGAAAGCTTATGTCGACTGCCTGATGGACCGAGGACGATGCACTCCTGATGGAAAGGCTCTTAAGGAAACTCTTCCTGACGCCTTAGAAAATGAATGCAGCAAATGCACAGAGAAGCAAAAGTCTGGCTCAGACAAAGTGATCAGGCACTT
